# Artificial intelligence applications in surgical education and training: a systematic review

**DOI:** 10.3389/frai.2026.1815315

**Published:** 2026-06-24

**Authors:** Talia Tene, Paulina Elizabeth Valverde Aguirre, Ángel Floresmilo Parreño Urquizo, Diego Fabián Vique López

**Affiliations:** 1 Department of Chemistry, Universidad Técnica Particular de Loja, Loja, Ecuador; 2 Facultad de Ciencias, Escuela Superior Politécnica de Chimborazo (ESPOCH), Riobamba, Ecuador; 3 Facultad de Salud Pública, Escuela Superior Politécnica de Chimborazo (ESPOCH), Riobamba, Ecuador

**Keywords:** AI, large language models, robotic surgery, simulation, surgical education, surgical training, systematic review

## Abstract

**Introduction:**

Surgical training is shifting toward scalable, data-driven education as operative complexity and patient safety expectations increase. AI can support objective feedback and cognitive guidance.

**Methodology:**

We conducted a PRISMA-guided systematic review using a PICO framework and searched PubMed, Scopus, and IEEE Xplore for peer-reviewed studies published between 2020 and 2025.

**Results:**

Of 1,109 records, 21 studies met the inclusion criteria. Included studies covered simulation-based, robotic, laparoscopic, and computer-assisted training using deep learning/computer vision, tutoring or predictive models, and language-based tools. Performance outcomes predominated (81.0%) over engagement-related outcomes (19.0%), and reported effects were mainly positive (76.2%) or increased (23.8%).

**Discussion:**

Evidence suggests near-term, task-specific gains when AI provides objective measurement and feedback, but comparability is limited by heterogeneous endpoints, small samples, and single-center designs.

**Conclusion:**

AI-enabled surgical education shows promise for objective assessment and adaptive instruction, but multicenter longitudinal studies with standardized metrics are still needed.

## 1 Introduction

Surgical education is evolving as traditional apprenticeship models are increasingly complemented by objective, scalable, and data-driven approaches (Scott et al., 2008). Modern surgical training demands objective, scalable, and data-driven methodologies to address the rising complexity of operative procedures and the imperative for heightened patient safety (Hussain et al., 2023; Levendovics et al., 2024). Artificial intelligence has emerged as an important technological approach in this context, enabling the analysis of large volumes of surgical data for educational assessment and feedback (Scott et al., 2008; Mansoor and Ibrahim, 2025).

The digitization of the surgical environment serves as the foundation for this evolution (Ashrafian et al., 2017). Operating rooms and simulation centers now function as high-fidelity data generators where every maneuver is recorded (Levendovics et al., 2024; Bludevich et al., 2024). The challenge lies in analyzing these multidimensional datasets, early attempts at automated assessment relied on basic motion metrics (Alshamrani et al., 2022), but these lacked the nuance required to evaluate clinical judgment or technical fluidity (Balch et al., 2024). Current advancements in deep learning architectures, particularly those utilizing convolutional neural networks and vision transformers (Khan et al., 2025), allow for the precise identification of surgical phases and tool-tissue interactions (Hussain et al., 2023; Kafai Golahmadi et al., 2021). These systems facilitate a move away from subjective expert evaluations toward standardized, quantitative performance metrics (Kankanamge et al., 2025).

Beyond simple detection, the integration of temporal modeling through architectures such as long short-term memory networks and spatiotemporal classifiers has expanded the ability to analyze skill acquisition (Schmidgall et al., 2024). Surgical proficiency is inherently dynamic (Stefanidis, 2010); it exists in the relationship between time and space (Keehner et al., 2006). Modern AI frameworks can now distinguish between the erratic movements of a novice and the precise, accurate gestures of an expert (Raptis et al., 2026). This capability may support personalized feedback loops that operate with reduced reliance on constant human supervision (Guni et al., 2024). AI-enabled educational systems may use these insights to tailor training activities to individual needs, supporting progression based on demonstrated competency rather than fixed training time (Raghavan et al., 2025).

The scope of surgical education is also expanding through the application of large language models and generative artificial intelligence (Karabacak et al., 2023). These tools are being integrated into the broader educational ecosystem to manage cognitive load and streamline administrative workflows (Sheikh and Fann, 2019). From the automated scoring of residency application materials to the generation of patient-specific educational resources (Kewalramani et al., 2026), AI acts as an augmentative layer that reduces the burden on faculty (Guerrero et al., 2022). The development of digital twins and physics-based neural networks allows for high-fidelity simulations where trainees can practice complex procedures in a risk-free virtual environment that mirrors real-world anatomical responses (Batool et al., 2026).

The use of these technologies requires careful consideration of interpretability and explainability (Raposo, 2025). For AI to be effectively integrated into a surgical curriculum (Roveta et al., 2025), the feedback provided must be interpretable and transparent. Educational frameworks are increasingly exploring how to incorporate these systems while preserving the central role of the human mentor (Marey et al., 2024). Such hybrid approaches aim to combine machine-assisted assessment with the essential human element of surgical mentorship (Dabbagh and Sabouri, 2025). The integration of artificial intelligence into surgical training may support more objective, competency-based progression (Guni et al., 2024; Ismail and Rahimi, 2025). Unlike traditional, time-bound training, AI-based platforms facilitate a detailed understanding of technical deficiencies. By identifying deviations in instrument trajectory or excessive force application (Yangi et al., 2025), these systems may help guide targeted feedback during training (Ismail and Rahimi, 2025). Machine learning approaches using eye tracking and biometric sensors may also help quantify fatigue and cognitive distraction during training and assessment (Dabbagh and Sabouri, 2025).

Another proposed advantage of these computational advances is the broader accessibility of standardized training support (Wah, 2025). High-quality mentorship is often restricted by geographical and institutional barriers (Awuah et al., 2024). AI-enhanced training modules may help address this gap by offering more standardized guidance in resource-limited settings (Loaiza-Bonilla et al., 2025). The use of neural machine translation and intelligent tutoring systems ensures that the latest surgical techniques and educational materials are accessible globally (Fazlolahi et al., 2022), regardless of language or location (Chang et al., 2020). This broader accessibility may be particularly relevant in settings with limited access to specialized surgical mentorship (Shahrezaei et al., 2024). Ethical and regulatory considerations remain paramount as these technologies become embedded in medical education (Jin et al., 2025). The management of surgical data requires robust frameworks to protect patient privacy and ensure data security (Jaime et al., 2023). As AI models take on a more prominent role in assessment, addressing algorithmic bias becomes an important priority (Roveta et al., 2025). Greater attention to dataset diversity is necessary to reduce the risk of reinforcing existing disparities in surgical training (Loftus et al., 2020). The ongoing dialogue between surgeons, ethicists, and computer scientists is necessary to establish guidelines that prioritize both innovation and professional integrity.

This work presents a systematic review of artificial intelligence applications in surgical education and training published between 2020 and 2025. This review examines how AI-based tools have been used and evaluated across surgical training settings involving medical students, surgical residents, fellows, and, when relevant, surgical nursing personnel. The study seeks to identify commonly reported pedagogical applications, evaluate the performance metrics used in the literature, and describe the technological and educational challenges associated with broader integration of these systems into surgical training programs. By consolidating these findings, this work provides an overview of the field and its current level of educational maturity.

## 2 Methodology

This systematic review follows a rigorous methodological framework to synthesize empirical evidence on artificial intelligence applications in surgical education and training, with emphasis on their educational use, technical validation, and reported impact on measurable learning outcomes. The study adheres to the PRISMA (Preferred Reporting Items for Systematic Reviews and Meta-Analyses) guidelines to ensure transparency and replicability (Liberati et al., 2009). The main objective is to evaluate empirical research focused on the design and effectiveness of AI-based educational interventions in various surgical modalities, including robotic, laparoscopic, and open surgery.

The literature search was conducted in major academic databases, such as PubMed, Scopus, and IEEE Xplore, seeking peer-reviewed evidence published between 2020 and 2025. Data extraction prioritized objective educational outcomes such as technical competence, diagnostic accuracy, and cognitive workload management. Evidence was synthesized descriptively based on study design, participant population, AI modality, educational context, and reported pedagogical outcomes. A formal risk-of-bias or study quality assessment tool was not applied. This decision reflects the heterogeneity of the included literature, which comprised technical development studies, validation studies, active training interventions, and needs-assessment or acceptability studies with non-uniform designs and outcomes. Study characteristics were summarized descriptively, and evidence maturity was interpreted according to study purpose, design, and educational deployment, distinguishing technical development or validation studies, needs-assessment or acceptability studies, and active or controlled training interventions. Accordingly, the review was intended to characterize and synthesize the current empirical literature in this emerging field rather than to produce pooled comparative estimates. The findings should therefore be interpreted as a descriptive synthesis of the available evidence, and the absence of formal quality appraisal limits confidence in cross-study comparisons and in the overall strength of the conclusions.

The review specifically examines a broad spectrum of AI architectures, from convolutional neural networks for task recognition to large language models for clinical reasoning. Studies were selected based on measurable pedagogical impact and/or technical validation relevant to surgical education. This approach allowed assessment of current technological trends, reported educational effects, and the maturity of AI-enabled training approaches in modern surgery.

### 2.1 Review design and application of the PRISMA and PICO frameworks

The methodological rigor, transparency, and reproducibility of this systematic review were ensured through adherence to the PRISMA guidelines. Title and abstract screening and full-text eligibility assessment were conducted collaboratively by the author team. Decisions regarding study inclusion were resolved through discussion and consensus. The PICO framework (Population, Intervention, Comparison, Outcome) served as the structural foundation for the study (Kloda et al., 2020). These established standards facilitated a comprehensive approach to identifying, selecting, and synthesizing relevant literature regarding the integration of artificial intelligence into surgical education, focusing on technical skill acquisition and pedagogical strategies across diverse clinical modalities (Table 1).

**Table 1:** PICO framework defining the core elements used to structure the research question and inclusion criteria for the systematic review on artificial intelligence in surgical education and training.

	Element	Description
P	Population	Medical students, surgical residents, specialized fellows, and surgical nursing personnel involved in surgical education and training.
I	Intervention	Educational and training programs, simulation platforms, or instructional strategies integrating AI architectures.
C	Comparison	Traditional surgical training, non-AI-based simulation, or standard human-led apprenticeship models.
O	Outcome	Measurable pedagogical metrics including technical proficiency, procedural accuracy, cognitive workload, and automated performance.

Using the PICO model, the research question was iteratively refined to align with the objectives of this review: to investigate the impact of artificial intelligence architectures on the technical and cognitive development of individuals involved in surgical education and training. The final research question guiding this review was:

“How has artificial intelligence been applied and validated within educational and training programs to improve the technical and cognitive development of individuals involved in surgical education across different surgical modalities?”

In this review, application refers to the reported educational use and empirical evaluation of AI-based tools within surgical training settings. Accordingly, this review focuses on how these technologies were used, tested, and evaluated in educational settings, rather than on long-term curriculum-level adoption or institutional integration. Based on this question and the PICO framework, a structured Boolean query was developed to systematically retrieve relevant literature. Keywords were selected that reflected the specific interventions, the target population, and the various surgical instruction formats. This strategy ensured the retrieval of high-fidelity evidence reflecting the state of the art in technology between 2020 and 2025. The final formulation of the search string is summarized in Table 2.

**Table 2:** Search queries and results by database for AI in surgical education (2020–2025).

Database	Query	Results
Scopus	(“AI” OR “Artificial intelligence”) AND (“Education” OR “Training”) AND “Surgery”	229
PubMed		381
IEEE Xplore		499

### 2.2 Database selection and search strategy

To ensure a comprehensive and multidisciplinary collection of articles, three major academic databases were selected for this review: SCOPUS, PubMed, and IEEE Xplore. Each database contributes a unique perspective at the intersection of healthcare, pedagogy, and emerging computational technologies.

**SCHEME 1:**
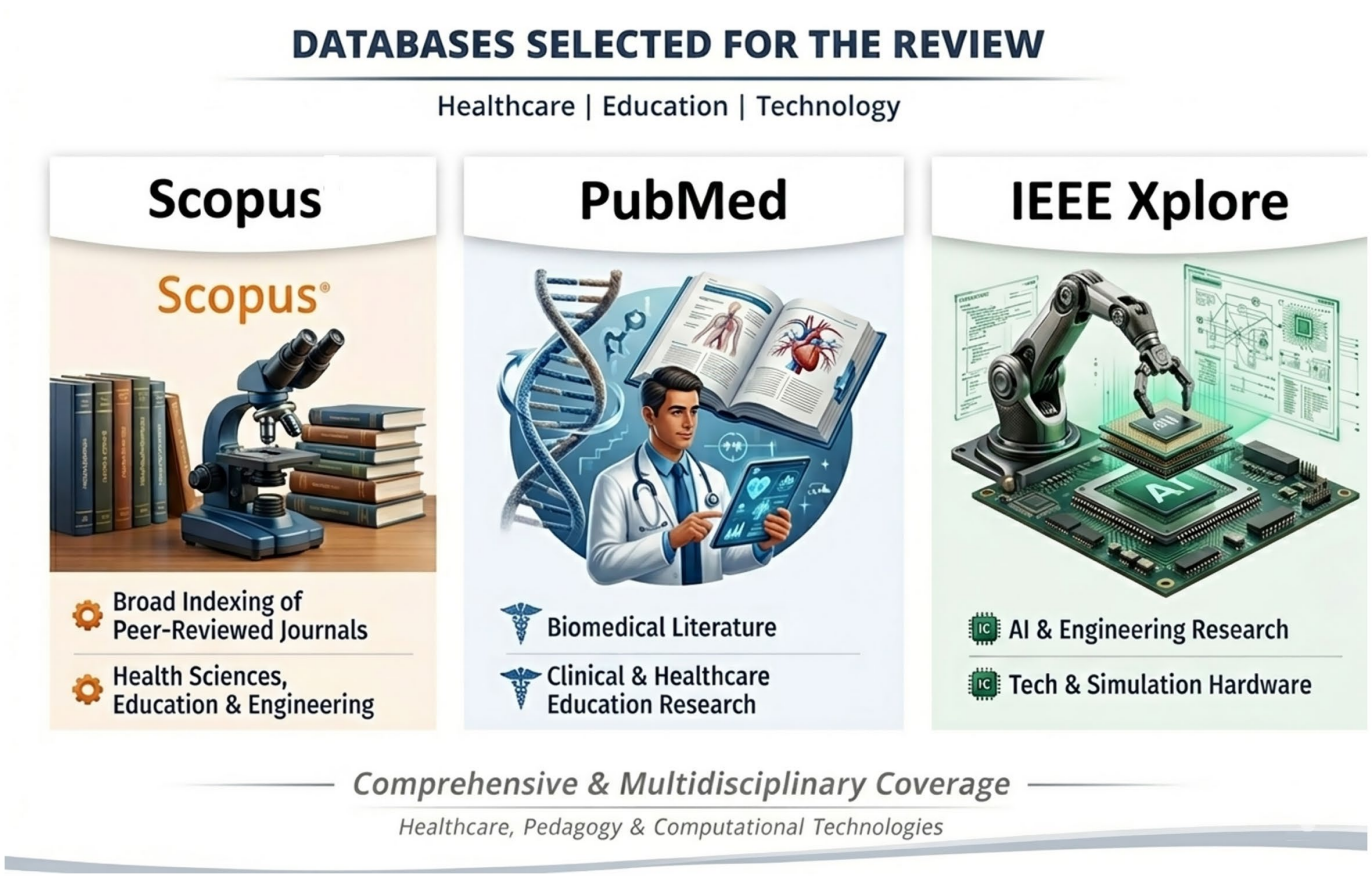
Databases selected for review (Scopus, PubMed and IEEE Xplore) and their complementary coverage in healthcare research, education and training of AI in relation to surgery. Image was created using ChatGPT 5.5 under Plus subscription.

The database search was conducted on 12 January 2026. The same Boolean search string was applied across SCOPUS, PubMed, and IEEE Xplore: (“AI” OR “Artificial intelligence”) AND (“Education” OR “Training”) AND (“Surgery”). The search was limited to studies published between 2020 and 2025 and to records in English. No final restriction by study design was imposed at the retrieval stage. Document-type exclusions and eligibility decisions were applied during the subsequent screening and full-text review stages, as shown in Scheme 1.

The Boolean formulation of the primary keywords (Table 2) targeted studies at the intersection of artificial intelligence and surgical training. Applying the same query across the three databases ensured consistency in retrieval while allowing comparison of source yield by platform. The 2020–2025 timeframe was selected to capture recent developments in generative artificial intelligence, vision transformers, and large language models during a period of rapid digital expansion in surgical education. The number of records retrieved from each database is summarized in Table 2.

The search strategy relies on a concise yet highly specific Boolean query. Given the high volume of AI-related publications in general healthcare, broader queries risked retrieving a significant number of non-educational or purely clinical articles. Limiting the search to this central intersection allowed the review to:

- Prioritize studies with explicit pedagogical or curricular content related to surgical skill acquisition.
- Minimize irrelevant matches from overlapping fields, such as general diagnostic radiology or pure algorithmic development without educational validation.
- Preserve the systematic integrity of the selection process by identifying studies most aligned with the established PICO framework.

**Figure 1:**
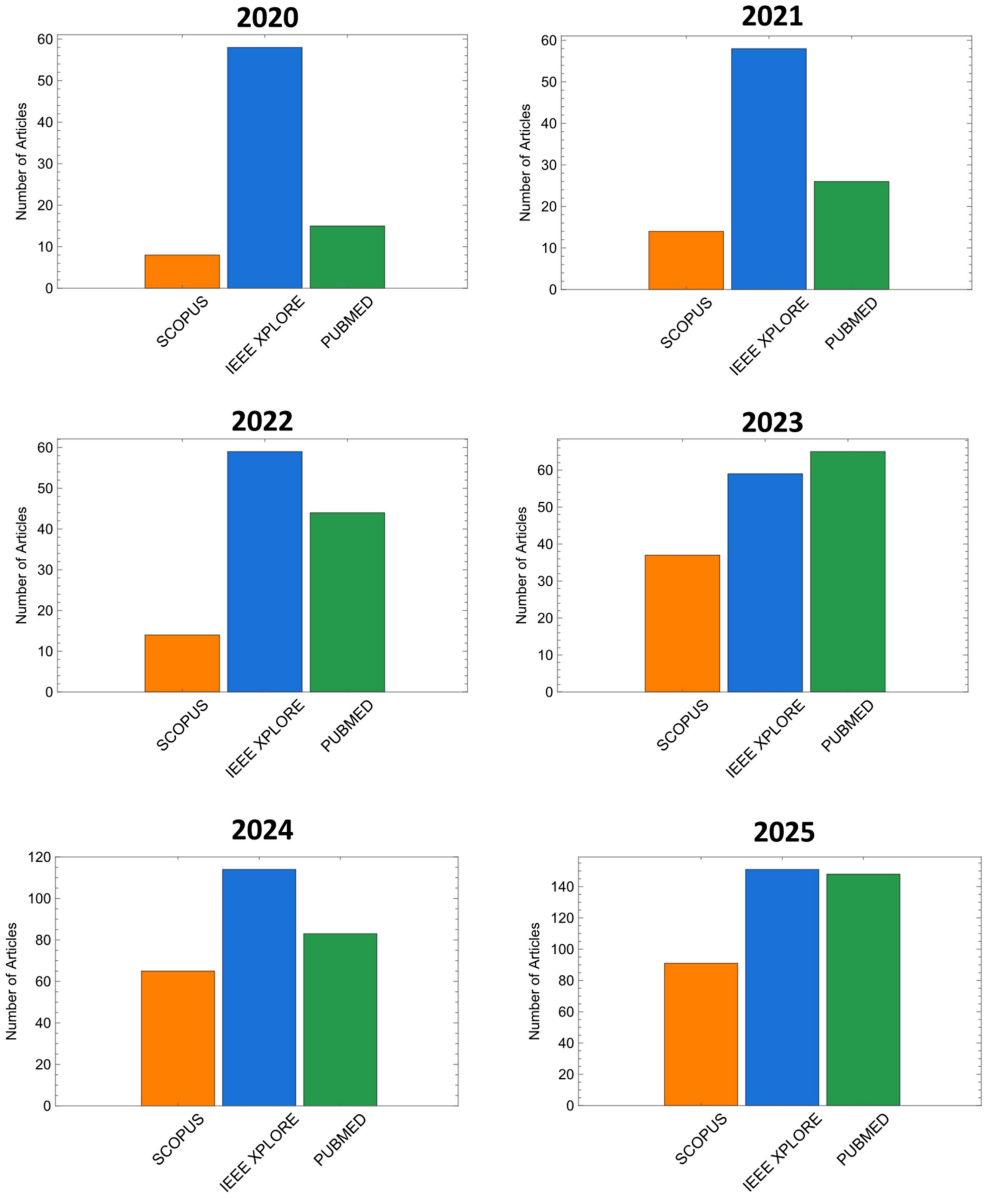
Chronological distribution of peer-reviewed publications across Scopus, PubMed, and IEEE Xplore databases (2020–2025), highlighting the surge in artificial intelligence research within surgical education and training.

The volume of retrieved publications reveals a trajectory of sustained growth, reflecting the maturation of artificial intelligence as a cornerstone of surgical pedagogy. As detailed in Figure 1, academic interest has evolved as follows:

Initial Period (2020–2022); during the early part of the decade, academic output was predominantly driven by the technical focus of IEEE Xplore, maintaining a stable output of approximately 58 to 59 annual articles. During this phase, PubMed demonstrated dynamic growth, nearly tripling its volume from 15 articles in 2020 to 44 in 2022. This shift indicates an early transition of algorithms from laboratory settings toward clinical educational validation.

Inflection Point (2023); this year represents a significant acceleration in source diversity. While IEEE Xplore maintained a consistent pace (59 articles), PubMed and SCOPUS experienced a notable surge. PubMed reached 65 articles, aligning biomedical literature growth with technical output, while SCOPUS more than doubled its previous year’s production, reaching 37 publications.

Massive Expansion Phase (2024–2025), the final biennium of the study demonstrates exponential growth. In 2024, total volume surged, with IEEE Xplore reaching 114 publications and PubMed rising to 83. This trend culminated in 2025, the year with the highest research activity in the entire period: IEEE Xplore led with 151 articles, followed closely by PubMed with 148 and SCOPUS with 91.

### 2.3 Identification

**Figure 2:**
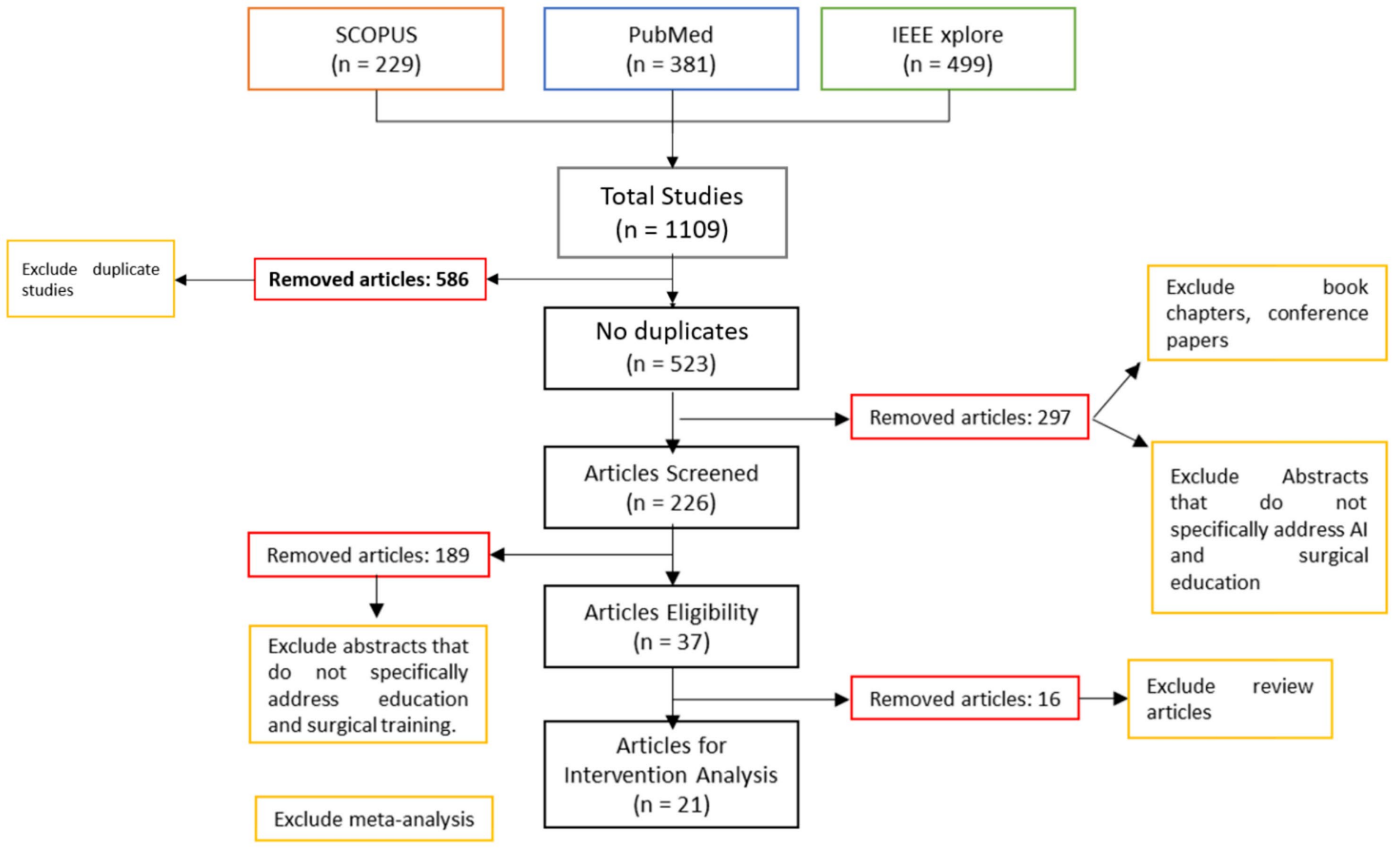
PRISMA flowchart showing the selection process of studies across four phases: identification, screening, eligibility, and inclusion.

The process of identifying and selecting studies for this systematic review was carried out in accordance with the PRISMA principles. Figure 2 illustrates in detail the different phases of the process, from initial identification to final inclusion of articles for analysis.

The identification phase commenced with a systematic search across three primary academic repositories to capture the intersection of artificial intelligence and surgical training. This initial query yielded a total of 1,109 potential records, distributed as follows: SCOPUS contributed 229 articles, PubMed provided 381, and IEEE Xplore registered the highest volume with 499 contributions. These figures reflect the multidisciplinary nature of the research, spanning biomedical sciences, clinical education, and technical engineering.

A critical step in this phase involved the elimination of duplicate records to ensure a clean dataset for subsequent screening. Utilizing automated reference management software followed by manual verification, 586 duplicates were identified and removed. This substantial reduction emphasizes the overlap between medical and technical databases in the context of digital surgery. Following the removal of these redundancies, a total of 523 unique records remained for preliminary evaluation. This phase was designed to be highly inclusive, ensuring that no emerging technological architectures or novel pedagogical strategies were overlooked during the initial harvest of scientific literature between 2020 and 2025.

### 2.4 Screened

The screening phase involved a two-tier evaluation of the 523 unique records remaining after duplicate removal to distill the literature down to studies with direct relevance to the research question. Initially, a formal exclusion of non-journal formats was conducted; 297 records were excluded because they were classified as book chapters or conference papers. This criterion was established to prioritize peer-reviewed journal articles that provide comprehensive methodologies and validated results, which are essential for a systematic synthesis of educational efficacy. Many of the excluded records focused on algorithmic development, general diagnostic radiology, or clinical outcomes without an instructional or training component. Following this screening step, 226 records remained for full-text eligibility assessment.

### 2.5 Eligibility

In the eligibility phase, the 226 full-text articles were assessed to confirm their alignment with the PICO framework established for this study. This stage was critical for differentiating between general mentions of AI in healthcare and substantive educational interventions. A significant portion of the literature was excluded during this deep dive; specifically, 189 articles were removed for failing to specifically address both education and surgical training. Many of these studies, while technically sound, focused on intraoperative decision support for attending surgeons rather than the training and skill acquisition phase for residents or students.

The eligibility criteria mandated the presence of measurable educational outcomes. Studies that were purely descriptive or lacked objective validation metrics were excluded. Following this intensive scrutiny, the pool was narrowed down to 37 eligible articles. This phase ensured that only eligible empirical studies were retained, focusing on reports that demonstrated a clear link between the educational application of AI architectures and measurable outcomes in surgical training across different operative modalities.

### 2.6 Inclusion

The final inclusion phase represented the last stage of study selection, focusing on study type and alignment with the predefined eligibility criteria. From the 37 articles deemed eligible, a further 16 records were excluded because they were identified as review articles rather than original empirical research. Furthermore, meta-analyses were excluded to prevent the duplication of primary data findings within this systematic review. This rigorous filtration ensured that the final dataset consisted of original empirical studies providing data on AI-enhanced surgical pedagogy.

A total of 21 original research articles were included in the final descriptive synthesis. These selected studies represent the state-of-the-art in surgical training between 2020 and 2025, covering a spectrum of architectures from convolutional neural networks to large language models. The selection process, as mapped in the PRISMA flow chart in Figure 2, guarantees that the resulting synthesis is based on a refined set of included empirical studies selected through the predefined eligibility criteria. These 21 studies provide the necessary empirical data to evaluate the impact of AI on technical skill development, procedural accuracy, and the overall modernization of the surgical curriculum.

## 3 Results

The final analysis focused on 21 studies describing specific artificial intelligence applications within the domain of surgical education and training. This final sample reflects an emerging and heterogeneous body of literature that includes technical development and validation studies, active training interventions, and needs-assessment or acceptability studies related to AI in surgical education. The evidence ranges from automated educational tools for medical students to sophisticated decision-support systems and predictive modeling for experienced surgeons. For interpretive clarity, these studies were not treated as equivalent educational interventions. Instead, the analysis was organized into two distinct analytical categories: (1) Educational Integration, in which AI was applied directly to teaching, training, feedback, or skill acquisition; and (2) Clinical-adjacent and administrative applications, in which AI supported planning, ergonomics, communication, selection processes, or broader educational access.

### 3.1 Evolution of AI in surgical pedagogy in cognitive instruction

Beyond its role as an analytical tool, artificial intelligence may also function as a cognitive scaffold by supporting perception, reducing uncertainty, and personalizing instruction. Table 3 details these interventions by AI/ML approach, target variables, and context, showing a progression from measurement-focused systems toward more adaptive instructional models and language-mediated educational support (Fukuta et al., 2025; Wang et al., 2025; Varman et al., 2025; Gomez et al., 2025; Chen et al., 2025; Giglio et al., 2025; Stone et al., 2025; Lee et al., 2025; Tharakan et al., 2024; Okumura et al., 2025; Becerik et al., 2025; Ma et al., 2024; Jackson et al., 2024; Nakao E. et al., 2025; Ozkan et al., 2025; Till et al., 2025; Checcucci et al., 2023; Fazlollahi et al., 2023; Moglia et al., 2022; Madani et al., 2022; Wu et al., 2020). An established research pattern focuses on quantifying the hidden cognitive states that dictate technical performance. In this paradigm, AI-enabled sensing—such as computer vision motion tracking for objective assessment of forceps manipulation (Fukuta et al., 2025)—infers trainee readiness and precision, allowing educators to pinpoint technical and cognitive bottlenecks invisible to traditional scoring. Furthermore, real-time pupillary dilation monitoring predicts perceived workload during robotic training; linking physiological signals to mental effort allows training designs to align task complexity with learner capacity (Wu et al., 2020). This measurement-first approach provides objective proxies for attentional control and skill retention in high-stakes environments (Fukuta et al., 2025; Wu et al., 2020).

**Table 3:** Synthesis of included studies evaluating the impact of artificial intelligence across surgical modalities, educational designs, and clinical specialties (2020–2025).

Reference	Intervention	AI/machine learning	Variable	Number of participants	Context
Fukuta et al. (2025)	Development and validation of an AI-assisted system for objective assessment of laparoscopic forceps manipulation during simulation training.	DeepLabCut (DLC); AI-based pose estimation; computer vision motion tracking	Performance	10 participants	Simulation Lab
Wang et al. (2025)	AI-assisted preoperative surgical simulation and intraoperative navigation using non-enhanced CT-based 3D reconstruction	Mamba-UNet + SegRefiner for image segmentation	Performance	163 participants	Computer-aided Surgery
Varman et al. (2025)	Using AI to score residency personal statements.	LLM (GPT-3.5)/Zero-shot Learning	Performance	668 Statements	Graduate Med Ed
Gomez et al. (2025)	Interpretable feedback on primitive actions.	Explainable AI (XAI)/Action Analysis	Performance	24 Participants	Simulation Lab
Chen et al. (2025)	Evaluation of translation tools for education access.	Neural Machine Translation (NMT)/LLMs	Performance	4 MT Tools	Global Education
Giglio et al. (2025)	AI tutoring alone vs. AI + personalized human instruction.	Intelligent Tutoring Systems (ITS)/DL	Performance	41 Students	Simulation Lab
Stone et al. (2025)	Instructorless training using XR headsets and DL.	Custom CNN (ResNet/VGG Architecture)	Performance	10 Students	Simulation (Phantom)
Lee et al. (2025)	Landmark and dissection plane detection for mastectomy.	Deep Learning (CNN/CenterNet)	Performance	10 Videos	Computer-aided Surgery
Tharakan et al. (2024)	Evaluating AI-generated FAQ responses vs. Google Search.	LLM (GPT-4/Generative Pre-training)	Performance	30 FAQs	Patient Education
Okumura et al. (2025)	Real-time recognition of surgical planes during GI.	Real-time Recognition Algorithms (CNN)	Engagement	56 Students	Clinical/Classroom
Becerik et al. (2025)	Quality assessment of AI patient materials (Rhinosinusitis).	LLM (GPT-4/Google Gemini)	Performance	20 FAQs	Online/Clinical
Ma et al. (2024)	Automated video feedback clips provided after suturing.	Vision Transformer (ViT)/Computer Vision	Performance	42 Novices	Simulation Lab
Jackson et al. (2024)	Survey on AI as assistive technology in the curriculum.	Not reported (Perception Survey)	Engagement	325 Students	University
Nakao E. et al. (2025)	AI-labeled videos highlighting anatomical structures.	Deep Learning (Object Detection)	Performance	158 Students	Medical School
Ozkan et al. (2025)	Assessing nursing student knowledge of robotic surgery.	Not reported (Qualitative Analysis)	Engagement	196 Students	University
Till et al. (2025)	Surgeon knowledge gap assessment via ESPES survey.	Not reported (Needs Analysis Survey)	Engagement	114 Surgeons	Professional Society
Checcucci et al. (2023)	Real-time system to detect and quantify bleeding.	Convolutional Neural Networks (CNN)	Performance	43 Procedures	Robotic Console
Fazlollahi et al. (2023)	AI-selected technical competencies vs. expert benchmarks.	Deep Learning (Classification Algorithms)	Performance	70 Students	Simulation Center
Moglia et al. (2022)	Predicting proficiency acquisition based on initial performance.	Ensemble Deep Neural Networks (DNN)	Performance	176 Students	Simulation Lab
Madani et al. (2022)	Identification of “Go/No-Go” zones for injury prevention.	Deep Learning (Semantic Segmentation)	Performance	290 Videos	Operating Room
Wu et al. (2020)	Real-time monitoring of pupil dilation to quantify workload.	Supervised ML Classifiers (SVM/RF)	Performance	8 Trainees	Simulation Lab

A second phase elevates AI from passive measurement to active, task-level guidance. Current evidence suggests that cognitive instruction succeeds when AI outputs are interpretable and synchronized with expert models. Explainable AI (XAI) systems now generate trainee-specific feedback by mapping primitive actions against expert benchmarks, facilitating deliberate practice through precise correction (Gomez et al., 2025). Similarly, deep learning architectures combined with 3D reconstruction from non-enhanced CT scans provide intraoperative navigation and preoperative simulation, bridging the gap between planning and execution (Wang et al., 2025). Data-driven tutoring architectures indicate that AI yields the greatest benefit when it amplifies human pedagogy (Giglio et al., 2025), while systems pairing extended reality (XR) with custom CNNs support autonomous guidance through an iterative loop of “perceive, classify, and correct” (Stone et al., 2025). Collectively, these studies suggest a shift toward “feedback during performance,” which helps accelerate learning and reduces the reinforcement of incorrect mental models (Wang et al., 2025; Gomez et al., 2025; Giglio et al., 2025; Stone et al., 2025).

A third phase emphasizes perceptual scaffolding, where learners are trained to “see what experts see.” AI-supported recognition systems enhance anatomical interpretation and the identification of correct dissection planes or landmarks in mastectomies, targeting the learner’s underlying judgment (Lee et al., 2025; Okumura et al., 2025). AI-based visualization tools and object detection highlight critical structures in clinical media, improving interpretive readiness (Nakao E. et al., 2025), while automated video feedback clips provide retrospective reinforcement after suturing (Ma et al., 2024). At the intraoperative level, semantic segmentation delineates risk boundaries, such as “Go/No-Go” zones, framing adverse events as failures in perception and positioning AI as a form of decision support (Madani et al., 2022). These modalities, including the real-time detection of bleeding (Checcucci et al., 2023) and the prediction of proficiency acquisition via ensemble deep neural networks (Moglia et al., 2022), align with competency-based education by treating situational awareness as a trainable outcome (Lee et al., 2025; Okumura et al., 2025; Ma et al., 2024; Nakao E. et al., 2025; Checcucci et al., 2023; Moglia et al., 2022; Madani et al., 2022).

The most recent expansion involves language-centered learning and institutional governance. Multilingual translation tools broaden global educational access (Chen et al., 2025), while Large Language Models (LLMs) like GPT-4 serve as interactive interfaces for generating FAQ responses and quality-assessing patient materials (Tharakan et al., 2024; Becerik et al., 2025). The application of LLMs to score residency personal statements introduces new efficiencies, though it necessitates a focus on zero-shot learning accuracy (Varman et al., 2025). At the curricular level, AI-selected technical competencies reshape training priorities (Fazlollahi et al., 2023). Finally, recent survey data and qualitative analyses reveal persistent gaps in AI literacy and knowledge of robotic surgery, suggesting that “AI competence” is now an essential educational endpoint (Jackson et al., 2024; Ozkan et al., 2025; Till et al., 2025).

**Figure 3:**
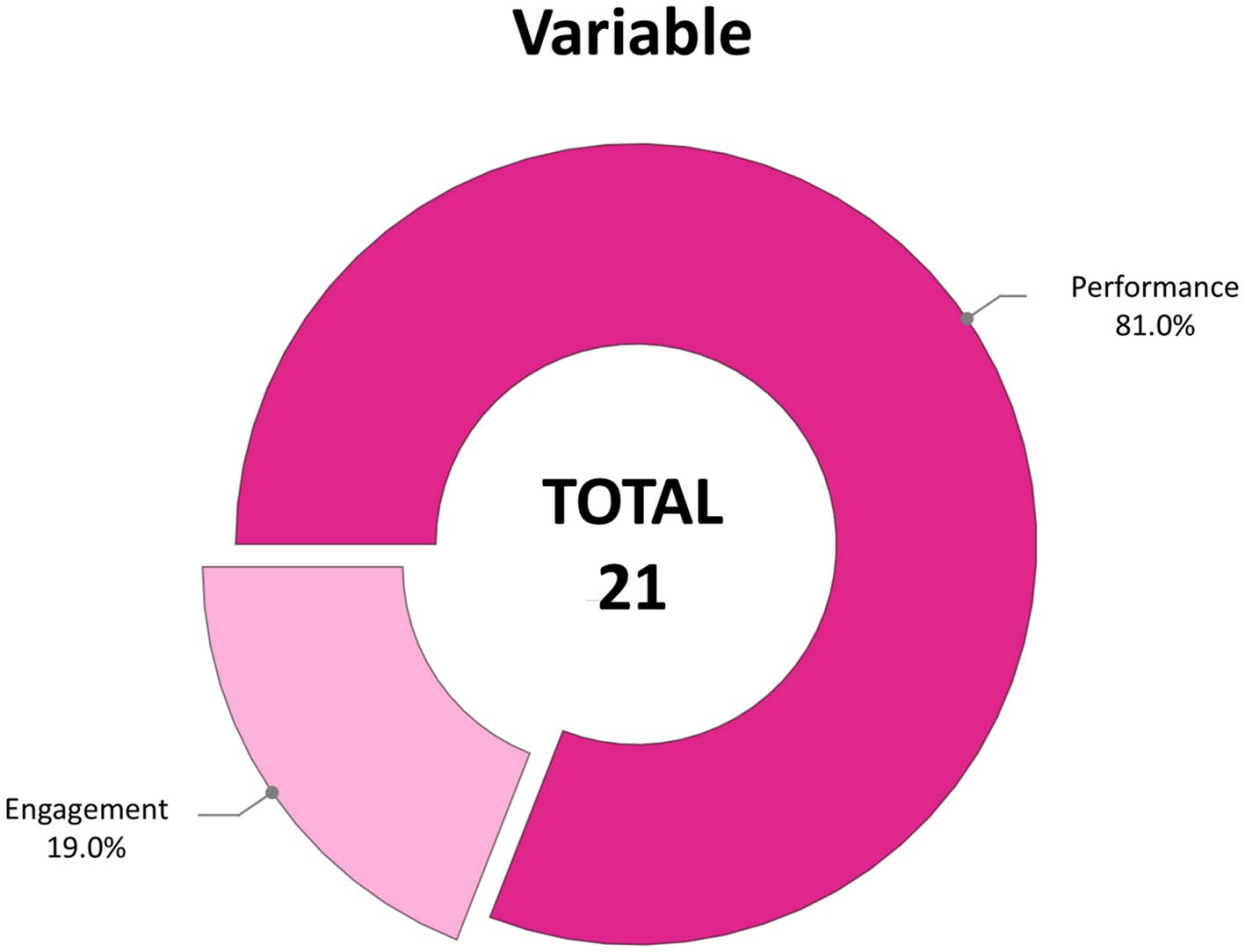
Distribution of variables assessed in AI-enabled surgical education and training studies.

Figure 3 shows that 81.0% of the included studies evaluated AI interventions using performance-related variables, whereas only 19.0% focused on engagement-related variables. This distribution highlights a strong emphasis on quantifiable, skill-adjacent outcomes—such as technical accuracy, proficiency classification, objective task metrics, anatomical recognition, and intraoperative decision support—over learner experience, attitudes, adoption readiness, or behavioral participation. Performance-oriented outcomes predominated in simulation, XR/VR, robotic, laparoscopic, and computer-assisted surgical contexts, where AI can be integrated directly into tasks and assessed through reproducible metrics such as automated scoring, predictive modeling of proficiency, segmentation-based safety zone identification, workload proxies, and real-time detection systems (Fukuta et al., 2025; Wang et al., 2025; Gomez et al., 2025; Chen et al., 2025; Giglio et al., 2025; Stone et al., 2025; Lee et al., 2025; Tharakan et al., 2024; Okumura et al., 2025; Becerik et al., 2025; Ma et al., 2024; Nakao E. et al., 2025; Checcucci et al., 2023; Moglia et al., 2022; Madani et al., 2022; Wu et al., 2020). By contrast, engagement-focused studies appeared more often in university or professional-society settings and primarily examined perceptions, knowledge gaps, readiness, and curricular expectations regarding AI-assisted surgery (Okumura et al., 2025; Jackson et al., 2024; Ozkan et al., 2025; Till et al., 2025). This imbalance suggests that the current literature is stronger in validating AI through objective performance measures than in assessing the human and institutional dimensions needed for sustained educational implementation.

### 3.2 AI in surgical education, trends in pedagogical utility, efficacy, and modality-specific outcomes

For the descriptive synthesis in Table 4, reported effects were classified as “Positive” when a study described favorable educational, technical, perceptual, or implementation-related findings without reporting a direct pre–post or comparative increase in a specific measured outcome. Effects were classified as “Increased” when a study reported a measurable gain or increase in a defined outcome, such as knowledge, performance score, detection capability, proficiency classification, or training-related competency. These labels were used only to summarize the reported direction of findings and should not be interpreted as standardized effect-size categories across studies.

**Table 4:** Synthesis of included studies evaluating the impact of artificial intelligence across surgical modalities, educational designs, and clinical specialties.

Reference	Surgical modality	Effect	Area	Design
Fukuta et al. (2025)	Laparoscopic surgical simulator training	Positive	Pediatric laparoscopic	Experimental feasibility study
Wang et al. (2025)	Thoracoscopic pulmonary segmentectomy	Positive	Thoracic surgery	Retrospective and prospective
Varman et al. (2025)	Admin	Positive	HR/GME	Retrospective
Gomez et al. (2025)	Training	Positive	Surgical Training	Human-AI Exp.
Chen et al. (2025)	Critical Care	Positive	Global Education	Multimodal Eval
Giglio et al. (2025)	Neuro-surgery	Positive	Neurosurgery	RCT
Stone et al. (2025)	Urology	Increased	Urology	Proof of Concept
Lee et al. (2025)	Robotic	Positive	Breast Oncology	Retrospective
Tharakan et al. (2024)	Robotic	Positive	Orthopedics	Cross-sectional
Okumura et al. (2025)	Laparoscopic	Increased	GI Surgery	Randomized Study
Becerik et al. (2025)	ENT	Positive	ENT	Comparative Eval
Ma et al. (2024)	Robotic (da Vinci)	Positive	Urology/Gen. Surg	Pilot RCT
Jackson et al. (2024)	General Med	Positive	Medical Education	Cross-sectional
Nakao E. et al. (2025)	Laparoscopic	Positive	GI Surgery	Cluster Quasi-RCT
Ozkan et al. (2025)	Nursing	Positive	Nursing	Mixed-methods
Till et al. (2025)	Endoscopic	Increased	Pediatric Surgery	Needs Analysis
Checcucci et al. (2023)	Robotic	Increased	Urology	Pilot Development
Fazlollahi et al. (2023)	Neuro-surgery	Increased	Neurosurgery	Cohort Study
Moglia et al. (2022)	Robotic	Positive	RAS Training	Predictive Modeling
Madani et al. (2022)	Laparoscopic	Positive	General Surgery	Diagnostic Validation
Wu et al. (2020)	Robotic	Positive	Ergonomics	Experimental

Positive = favorable reported finding without a directly quantified increase; Increased = measurable gain or increase in a defined outcome. Categories indicate reported direction, not standardized effect size.

Table 4 shows a consistent shift from traditional apprenticeship toward personalized learning cycles in which AI captures performance signals, provides targeted feedback, and supports iterative practice. This is explicit in systems designed for automated, user-specific feedback during skill acquisition (Gomez et al., 2025) and in AI-enabled tutoring frameworks that augment human instruction to improve simulation performance and skill transfer (Giglio et al., 2025). AI-based approaches increasingly aim to reduce subjectivity by providing standardized scoring or competency signals—whether in experimental feasibility studies for pediatric laparoscopic training (Fukuta et al., 2025), retrospective evaluations of thoracoscopic pulmonary segmentectomy (Wang et al., 2025), or competency design within simulation curricula (Fazlollahi et al., 2023). In parallel, AI is being used to expand access to education, which is particularly relevant for global or distributed training programs (Chen et al., 2025). In this sense, “utility” is not only measured as performance change, but also as scalability, accessibility, and standardization of training inputs.

The clearest efficacy pattern appears when AI is tied to specific tasks with measurable endpoints and actionable feedback. AI-driven video feedback improved novice robotic suturing performance in a controlled training paradigm (Ma et al., 2024), while in neurosurgical simulation, combining AI with personalized expert instruction produced superior outcomes versus intelligent tutoring alone (Giglio et al., 2025). This reinforces the principle that AI is most effective when it operates as a precision amplifier of instruction. Furthermore, the integration of AI in specialized fields—such as breast oncology (Lee et al., 2025), orthopedics (Tharakan et al., 2024), and ENT (Becerik et al., 2025)—demonstrates a positive impact on modality-specific training, while real-time monitoring of pupil dilation provides a measurable gain in understanding surgical ergonomics (Wu et al., 2020).

A second, emerging efficacy signal is the use of AI to predict learning trajectories, enabling differentiated training pathways. Deep learning ensembles have been used to predict proficiency acquisition rates in robot-assisted surgery (RAS), supporting adaptive curricula that allocate coaching intensity based on predicted needs (Moglia et al., 2022). In the context of gastrointestinal surgery, AI-labeled videos and real-time recognition systems have shown a measurable increase in learning engagement and anatomical identification (Okumura et al., 2025; Nakao E. et al., 2025).

While AI education has often centered on robotic video, the included studies demonstrate a broadening scope across diverse modalities. ML models have successfully addressed the objectivity gap in laparoscopic and endoscopic skills assessment (Till et al., 2025; Madani et al., 2022), and even in general medical or nursing curricula where AI serves as an assistive technology or a knowledge assessment tool (Jackson et al., 2024; Ozkan et al., 2025). Additionally, the development of pilot systems to detect and quantify bleeding in robotic consoles illustrates how AI can target high-stakes clinical outcomes within training environments (Checcucci et al., 2023). This diversification is relevant pedagogically because it supports standardized assessment at scale, a persistent limitation in technical training compared with instrumented simulation.

**Figure 4:**
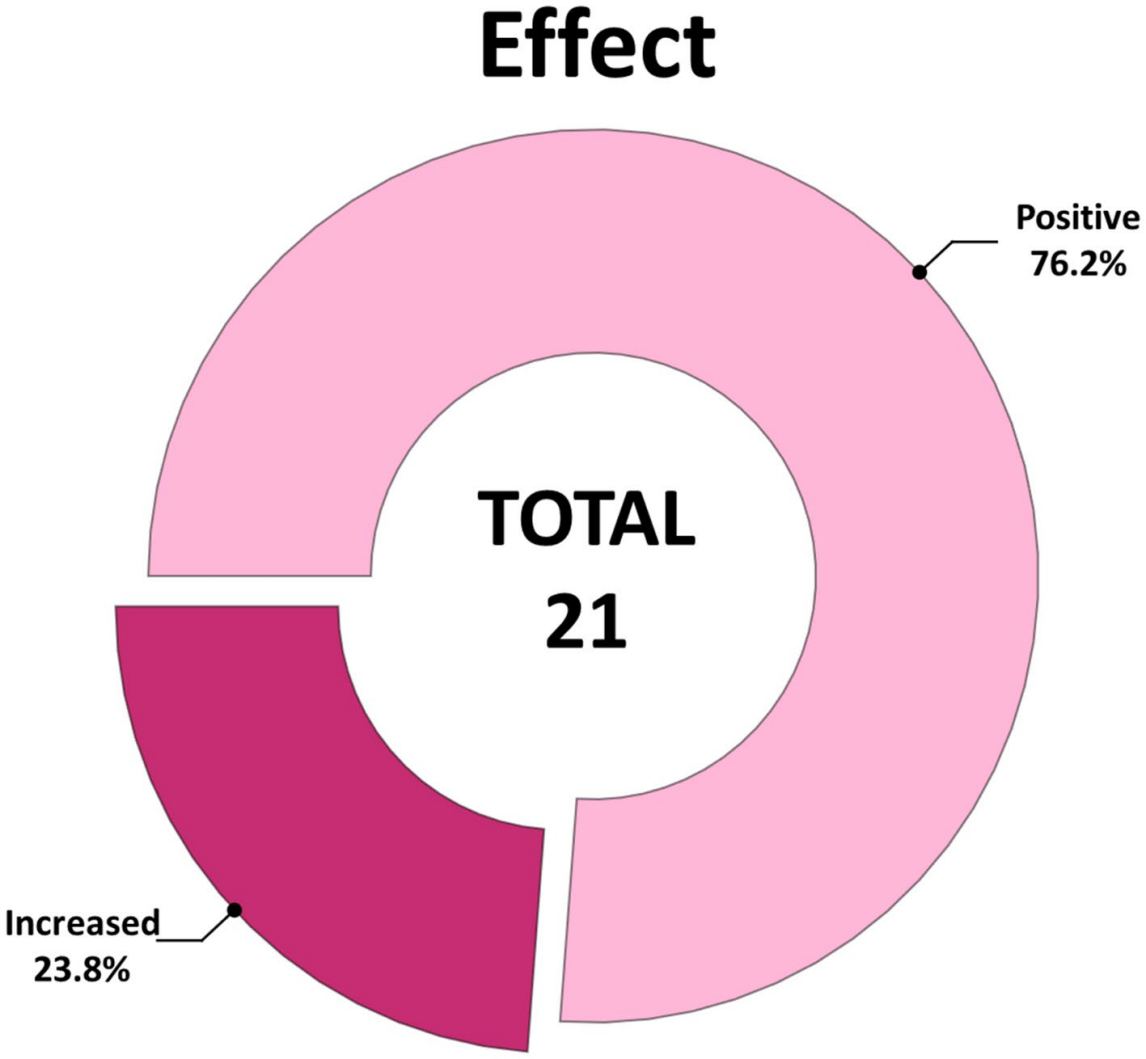
Distribution of reported effects of AI interventions in surgical education and training.

As shown in Figure 4, 76.2% of the studies reported a positive effect, whereas 23.8% reported an increased effect. The Positive category encompassed the broadest range of surgical education contexts, including simulation- and VR-based learning, open and minimally invasive environments, robotic training, and AI-based educational support tools, suggesting that AI was most often associated with beneficial educational use across multiple modalities (Fukuta et al., 2025; Wang et al., 2025; Varman et al., 2025; Gomez et al., 2025; Chen et al., 2025; Giglio et al., 2025; Lee et al., 2025; Tharakan et al., 2024; Becerik et al., 2025; Ma et al., 2024; Jackson et al., 2024; Nakao E. et al., 2025; Ozkan et al., 2025; Moglia et al., 2022; Madani et al., 2022; Wu et al., 2020). By contrast, the Increased category appeared more often in studies emphasizing measurable improvements in outcomes, capability, or competency-related assessment criteria, commonly linked to technical performance, anatomical recognition support, or AI/ML training needs rather than general perceptions of educational utility (Stone et al., 2025; Okumura et al., 2025; Till et al., 2025; Checcucci et al., 2023; Fazlollahi et al., 2023).

Although this distribution suggests an overall favorable direction of effect within the dataset, it should be interpreted cautiously. These categories reflect reported direction of effect rather than directly comparable effect magnitude across studies. In addition, the absence of neutral or negative labels may reflect publication bias and heterogeneous reporting, including inconsistent reporting of intervention duration and variation in how individual studies defined an “effect.”

**Figure 5:**
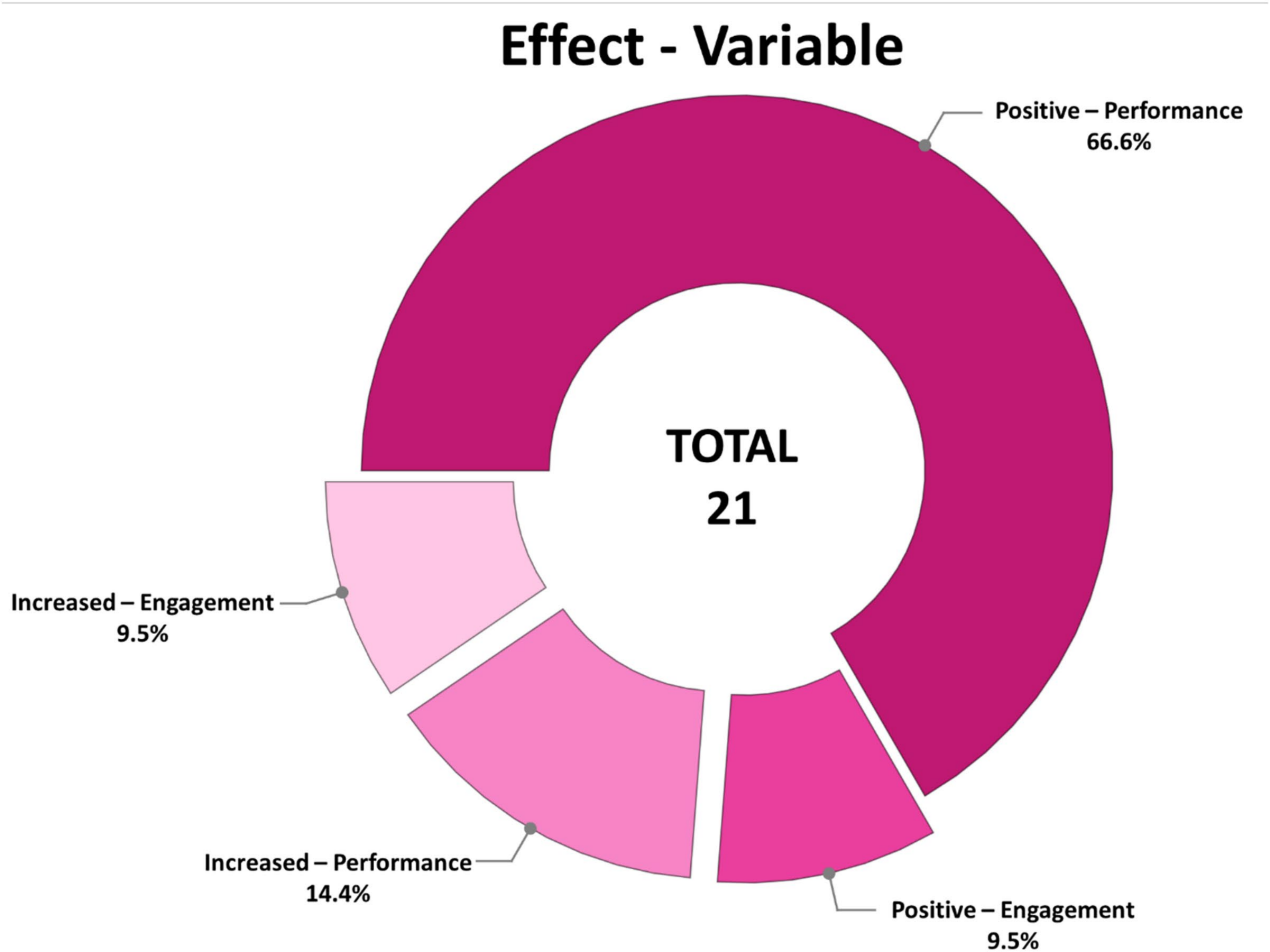
Distribution of reported effect and variable across included studies.

The analysis of effect types and variables reveals a research landscape heavily favoring technical metrics over behavioral integration. Figure 5 further clarifies the imbalance between technical and engagement-oriented evidence by showing that “Positive–Performance” was the dominant category, accounting for 66.6% of the dataset. These studies reported favorable findings through objective skill benchmarks, automated classification, anatomical recognition, safety-zone delineation, and cognitive workload proxies (Fukuta et al., 2025; Wang et al., 2025; Varman et al., 2025; Gomez et al., 2025; Chen et al., 2025; Giglio et al., 2025; Stone et al., 2025; Lee et al., 2025; Tharakan et al., 2024; Becerik et al., 2025; Ma et al., 2024; Nakao E. et al., 2025; Moglia et al., 2022; Madani et al., 2022; Wu et al., 2020). An additional “Increased–Performance” category represented 14.4% of the literature and was also centered on measurable gains in trainee capability or functional proficiency, even when broader descriptors of improvement were used (Stone et al., 2025; Checcucci et al., 2023; Fazlollahi et al., 2023). Together, these findings indicate that the literature is most developed in areas where AI can be linked to observable task performance and technical assessment.

By contrast, engagement-based findings were less frequent and more variably framed. “Positive–Engagement” (9.5%) captured learner perceptions, readiness, and institutional acceptance, primarily in university-based settings (Jackson et al., 2024; Ozkan et al., 2025). The remaining “Increased–Engagement” category (9.5%) included needs assessments or randomized educational interventions in which knowledge gaps and interaction quality were prioritized over psychomotor speed or accuracy (Okumura et al., 2025; Till et al., 2025). This pattern suggests that current evidence is stronger in demonstrating technical and performance-linked value than in addressing the behavioral, institutional, and adoption-related dimensions required for sustained educational implementation. For this reason, broader evaluation frameworks that incorporate engagement alongside technical outcomes remain necessary, particularly for language-mediated tools, translation systems, and curriculum-level applications (Varman et al., 2025; Chen et al., 2025; Okumura et al., 2025; Jackson et al., 2024; Till et al., 2025).

### 3.3 Implementation, assessment tools, and real-world limitations in AI-based surgical education

Evidence summarized in Table 5 indicates that AI systems for surgical training reside primarily within the developmental or skill-acquisition stages. This trend reflects a field focused on rapid prototyping and technical validation, though it has yet to consolidate clear pathways for routine curricular integration. The data show that educational impact was operationalized through a heterogeneous set of assessment instruments—ranging from objective kinematics and Dice Similarity Coefficient (DSC) metrics to psychometric surveys—which complicates cross-study comparability (Fukuta et al., 2025; Wang et al., 2025; Varman et al., 2025; Gomez et al., 2025; Chen et al., 2025; Giglio et al., 2025; Stone et al., 2025; Lee et al., 2025; Tharakan et al., 2024; Okumura et al., 2025; Becerik et al., 2025; Ma et al., 2024; Jackson et al., 2024; Nakao E. et al., 2025; Ozkan et al., 2025; Till et al., 2025; Checcucci et al., 2023; Fazlollahi et al., 2023; Moglia et al., 2022; Madani et al., 2022; Wu et al., 2020). Given this heterogeneity, evidence maturity was interpreted according to study purpose and educational deployment. Technical development and validation studies were considered preliminary feasibility evidence, as they mainly assessed model performance, measurement accuracy, or system functionality. Needs-assessment, survey, and acceptability studies were interpreted as implementation-readiness evidence because they identified perceptions, knowledge gaps, adoption barriers, or curricular expectations without directly measuring skill acquisition. Active training interventions provided early educational evidence by evaluating learner performance under structured training conditions, usually in simulation or controlled environments. Among these, controlled or randomized training studies represented the highest relative maturity within the included literature, although their interpretation remained limited by small samples, single-center settings, brief exposure periods, retrospective data, and limited follow-up.

**Table 5:** Implementation stage, assessment instruments, training deployment, reported barriers, and study limitations across AI-enabled surgical education and training interventions.

Reference	Stage	Assessment tools	Training/education	Barriers	Limitations
Fukuta et al. (2025)	Skill Development	Laparoscopyboxx Pro simulator	Training	Keypoint occlusion, motion blur from rapid movements.	Accuracy remains insufficient for operational use.
Wang et al. (2025)	Development	LungDimensionGo V1.0	Training	Dependence on annotated data limitations	AI model requires broader validation.
Varman et al. (2025)	Development	Weighted Kappa	Education	Potential algorithmic bias	Subjective text
Gomez et al. (2025)	Skill Development	Path length; Action freq	Training	Complexity of interpretation	Non-clinical setting
Chen et al. (2025)	Development	Likert (Fluency/Accuracy)	Education	Nuanced terminology	Limited languages
Giglio et al. (2025)	Skill Development	NeuroVR Simulator Metrics	Training	High hardware cost	Single-center
Stone et al. (2025)	Skill Development	ML Accuracy Classification	Training	Specialized hardware cost	Small pilot sample
Lee et al. (2025)	Development	DSC/HD metrics	Education	Lack of haptics	Single-center
Tharakan et al. (2024)	Development	Likert Scale	Education	AI hallucinations	Small FAQ sample
Okumura et al. (2025)	Skill Development	Questionnaire (Interest)	Training	High cognitive load	Small sample size
Becerik et al. (2025)	Development	DISCERN/EQIP scores	Education	LLM quality variance	Small question set
Ma et al. (2024)	Skill Development	AI-based needle handling metrics	Training	Acceptance of automated feedback	Small sample; single session
Jackson et al. (2024)	Development	Semi-structured Survey	Education	Ethical/Privacy concerns	Self-reported data
Nakao E. et al. (2025)	Skill Development	Anatomic Knowledge Test	Training	Anatomical complexity	Single-center
Ozkan et al. (2025)	Development	Survey/Focus Groups	Education	Knowledge gaps in robotics	Single-center
Till et al. (2025)	Development	ESPES AI Survey	Education	Poor knowledge base	Low response rate
Checcucci et al. (2023)	Development	AUC (0.88)	Education	Blood obscuring camera lens	Single-center
Fazlollahi et al. (2023)	Skill Development	VR Metrics (Force, Speed)	Training	Skill degradation (non-AI)	Secondary analysis
Moglia et al. (2022)	Skill Development	dV-Trainer Metrics	Training	High data requirement for DNN	Single-simulator validation
Madani et al. (2022)	Development	mAP/F1-score	Education	Lighting/Anatomy artifacts	Retrospective data
Wu et al. (2020)	Skill Development	NASA-TLX/Eye-tracker	Training	Hardware intrusiveness	Very small sample (n = 8)

Research involving active training prioritized technical results using quantitative tools such as simulator-derived kinematics, accuracy-based classification, and cognitive workload proxies like NASA-TLX paired with eye-tracking (Wang et al., 2025; Gomez et al., 2025; Giglio et al., 2025; Ma et al., 2024; Nakao E. et al., 2025; Fazlollahi et al., 2023; Moglia et al., 2022; Wu et al., 2020). Newer interventions have also introduced specialized platforms such as the Laparoscop-box Pro simulator for pediatric training (Fukuta et al., 2025) and LungDemeonGo V1.0 for thoracic surgery development (Wang et al., 2025). Conversely, studies focusing on readiness, acceptability, and institutional governance used Likert scales, focus groups, content quality tools such as DISCERN, or weighted kappa for text analysis (Varman et al., 2025; Chen et al., 2025; Tharakan et al., 2024; Becerik et al., 2025; Jackson et al., 2024; Ozkan et al., 2025; Till et al., 2025; Checcucci et al., 2023; Madani et al., 2022). These non-training designs support implementation logic but do not directly quantify the acquisition of new surgical skills. Accordingly, survey-based, administrative, language-mediated, and technical validation studies were interpreted as complementary but non-equivalent forms of evidence relative to active educational interventions.

Barriers documented in Table 5 help explain why translation remains uneven across modalities. Technical and environmental constraints—including keypoint occlusion and motion blur from rapid movements (Fukuta et al., 2025), lighting artifacts, and visual occlusion from bleeding—can degrade model performance and instructional reliability (Wang et al., 2025; Checcucci et al., 2023; Madani et al., 2022). Dependence on large annotated datasets also remains a critical hurdle for model validation (Wang et al., 2025; Moglia et al., 2022). Computational and usability barriers, including high hardware costs and limited haptic feedback, may restrict scalability and learner acceptance (Giglio et al., 2025; Stone et al., 2025; Lee et al., 2025; Wu et al., 2020). In language-mediated tools, LLM hallucinations and limited language coverage introduce educational risks that require expert verification (Chen et al., 2025; Tharakan et al., 2024; Becerik et al., 2025).

Methodological limitations, including small sample sizes, single-center designs, brief exposure periods, and retrospective data, restrict the generalizability of these findings (Fukuta et al., 2025; Gomez et al., 2025; Giglio et al., 2025; Ma et al., 2024; Jackson et al., 2024; Checcucci et al., 2023; Madani et al., 2022; Wu et al., 2020). While AI-enabled pedagogical systems show promise, broader educational validity requires standardized metrics and stronger links between algorithmic benchmarks, educationally relevant performance, and transfer of clinical skills to the operating room.

**Figure 6:**
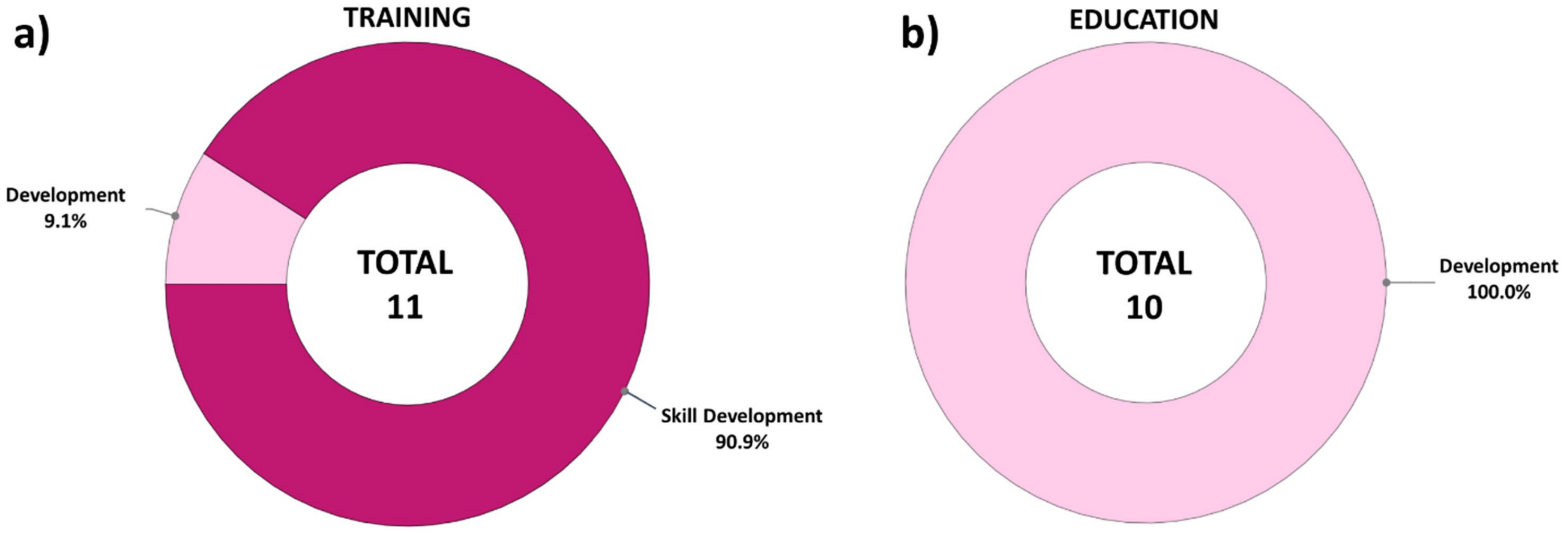
Stage distribution by deployment domain: **(a)** training-focused studies and **(b)** education-focused studies.

Figure 6 categorizes the included studies according to implementation stage and deployment domain, using the classifications reported in Table 5. Figure 6a shows the distribution among training-focused studies: Skill Development accounted for 90.9%, while Development accounted for 9.1%. Figure 6b shows the distribution among education-focused studies, all of which were classified as Development. This pattern is consistent with the findings summarized in Table 5, where training-focused studies were mainly linked to skill acquisition, simulator-based assessment, and performance measurement, whereas education-focused studies were primarily associated with tool development, technical validation, administrative applications, patient-education tools, surveys, and readiness assessments. The concentration of studies in these early stages indicates that AI-enabled surgical education remains largely pre-implementation, with most studies supporting technical maturation or early educational application rather than routine curricular integration.

This pattern is reinforced by the settings and barriers reported across the included studies. Even when active training was present, interventions were typically conducted in simulation laboratories or single-center environments with small samples and brief exposure periods, limiting generalizability and slowing progression toward long-term, multi-site adoption (Fukuta et al., 2025; Gomez et al., 2025; Giglio et al., 2025; Ma et al., 2024; Checcucci et al., 2023; Wu et al., 2020). Reported barriers further constrained this transition, including data quality issues, anatomical variability, specialized hardware requirements, and the absence of haptic feedback. Language-mediated tools introduced additional concerns related to translation quality, semantic reliability, and the risk of LLM hallucinations, while ethical and privacy considerations added further complexity to implementation in educational settings (Wang et al., 2025; Giglio et al., 2025; Stone et al., 2025; Lee et al., 2025; Tharakan et al., 2024; Becerik et al., 2025; Jackson et al., 2024; Checcucci et al., 2023; Madani et al., 2022).

## 4 Discussion

### 4.1 General impact and limitations of the evidence in AI-based surgical education and training

Synthesized evidence suggests that AI-enabled approaches in surgical education predominantly correlate with favorable results. Most research reports effects categorized as positive or increased across diverse modalities, from pediatric laparoscopic simulators to thoracic surgery platforms (Fukuta et al., 2025; Wang et al., 2025; Giglio et al., 2025). This trend aligns with a field actively validating tools designed to enhance learning efficiency, standardize assessment, and minimize feedback variability (Gomez et al., 2025; Ma et al., 2024). However, this consistency warrants careful interpretation because “effectiveness” is operationalized through highly heterogeneous metrics. Research variables range from objective task kinematics and classifier accuracy—such as the Dice Similarity Coefficient (DSC) or F1-score—to knowledge tests and survey-derived perceptions, complicating direct comparisons and obscuring the true magnitude of educational benefit (Wang et al., 2025; Jackson et al., 2024; Madani et al., 2022).

**SCHEME 2:**
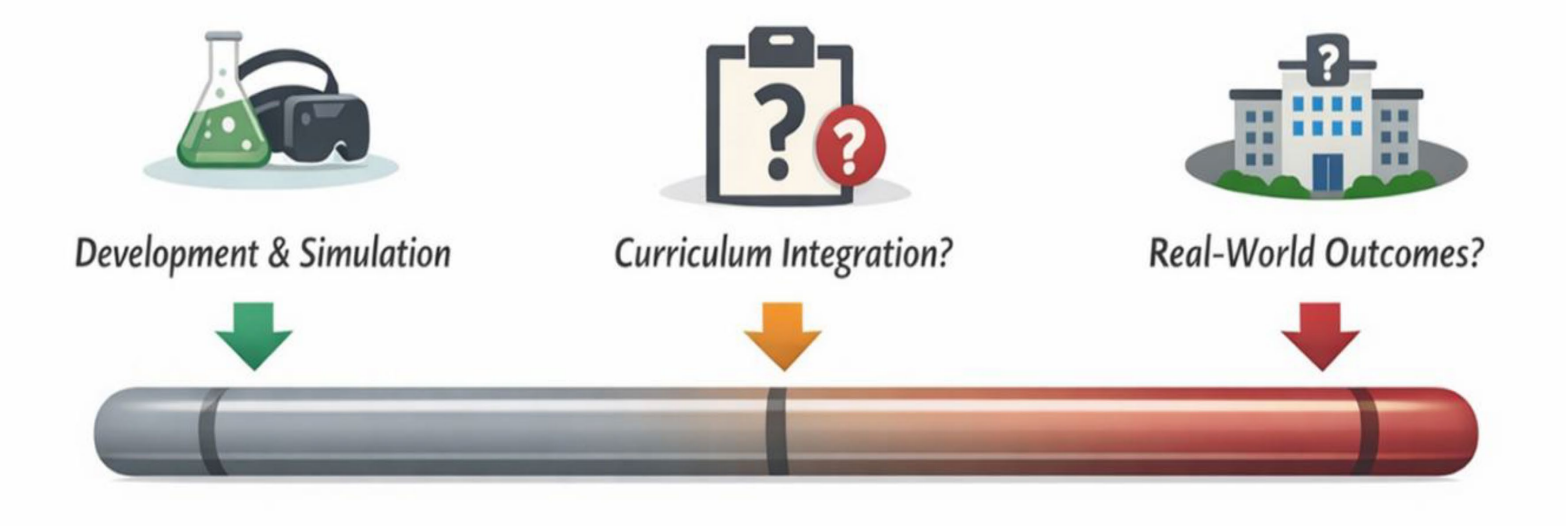
Evidence maturity pathway for AI in surgical education and training. Image was created using ChatGPT 5.5 under Plus subscription.

A primary challenge in defining effectiveness is the disproportionate focus on short-term performance metrics over long-term behavioral integration. Performance is typically measured in controlled environments—such as simulation labs or curated video datasets—where researchers can reliably detect improvements in accuracy, time, and proficiency classification (Fukuta et al., 2025; Gomez et al., 2025; Wu et al., 2020). This controlled setting can unintentionally inflate the effectiveness profile; gains are easier to demonstrate when tasks are standardized and environmental confounders, such as motion blur or lighting artifacts, are minimized (Fukuta et al., 2025; Ma et al., 2024; Moglia et al., 2022; Madani et al., 2022). In contrast, factors critical to real-world impact—including sustained engagement, trust calibration, and equitable access—remain under-measured and often rely on subjective self-reports (Jackson et al., 2024; Till et al., 2025) (Scheme 2).

The correlation between effect types and primary variables reinforces this imbalance. Most studies report positive results through performance outcomes, while engagement data represents a small minority and often features mixed framing, such as readiness signals or variable perceptions of utility (Okumura et al., 2025; Jackson et al., 2024; Ozkan et al., 2025; Till et al., 2025). Consequently, the current literature best supports claims regarding near-term, task-specific gains—such as improved simulation metrics in robotic suturing or enhanced perceptual guidance in mastectomies—rather than broad educational transformation (Lee et al., 2025; Ma et al., 2024; Moglia et al., 2022). While AI tools show promise for targeted competency components, the evidence remains insufficient to support conclusions about longitudinal competency progression or the transfer of skills to the operating room (Giglio et al., 2025; Checcucci et al., 2023).

The maturity of the current dataset further constrains these findings. In most included studies, AI-based tools were evaluated in restricted settings, often before routine curricular integration. Brief exposure periods and limited follow-up suggest that reported improvements may reflect short-term familiarization rather than durable learning (Giglio et al., 2025; Wu et al., 2020). In addition, the frequent use of single-center datasets and small participant samples ($n \leq 10$ in several technical validations) reduces external validity, particularly because AI performance varies across institutional workflows and imaging conditions (Fukuta et al., 2025; Wang et al., 2025; Stone et al., 2025; Checcucci et al., 2023).

Taken together, these characteristics indicate that the current literature supports the feasibility and early educational value of AI-enabled interventions more strongly than their long-term implementation. Importantly, improvements in technical accuracy or simulation-based performance should not be interpreted as direct evidence of clinical benefit, as most studies did not evaluate patient-level outcomes. Although several studies reported favorable effects where feedback was directly tied to specific training tasks (Fukuta et al., 2025; Gomez et al., 2025; Ma et al., 2024), stronger evidence is still needed to demonstrate skill retention, transfer to clinical practice, and sustained adoption across diverse educational environments (Giglio et al., 2025; Checcucci et al., 2023).

### 4.2 Evaluation focused on measuring adaptive and cognitive instruction on surgical education

**SCHEME 3:**
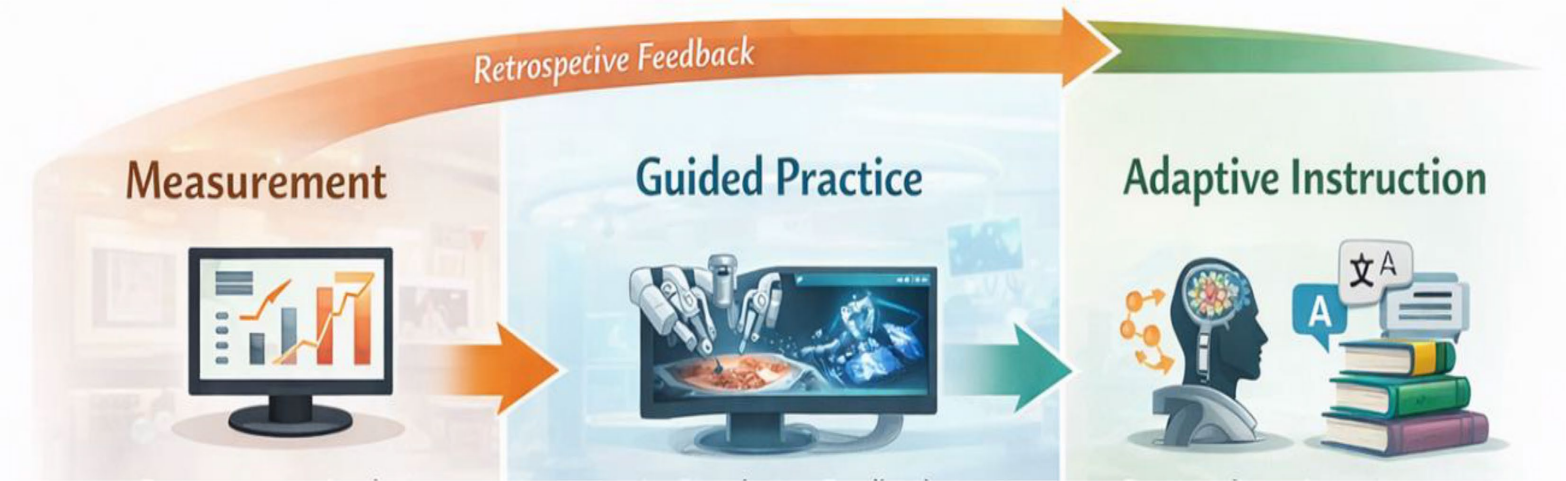
Pedagogical trajectory of AI-enabled surgical education. Image was created using ChatGPT 5.5 under Plus subscription.

The field is shifting from retrospective performance auditing toward systems that provide cognitive support throughout the learning process. These frameworks support trainees in perception, interpretation, and decision-making within clinically relevant contexts, rather than focusing solely on task execution (Okumura et al., 2025; Madani et al., 2022). This evolution manifests through the integration of AI outputs directly into the instructional loop—comprising execution, feedback, and targeted repetition—moving beyond post-hoc technical benchmarking (Fukuta et al., 2025; Gomez et al., 2025; Giglio et al., 2025). AI functions as an instructional co-processor, with its strongest applications appearing where objective signal capture is feasible and feedback aligns closely with learner actions (Giglio et al., 2025; Ma et al., 2024) (Scheme 3).

In simulation and XR/VR environments, this progression is reflected in the use of AI to structure guided practice. Platforms like the Laparoscop-box Pro simulator utilize AI to generate authentic training scenes and couple simulated experiences to measurable performance indicators, supporting deliberate practice and competency-based progression (Fukuta et al., 2025; Stone et al., 2025). These contexts provide a setting in which the educational utility of AI can be evaluated precisely, facilitating the iterative refinement of instructional logic while mitigating the variability inherent in live clinical training (Fukuta et al., 2025; Stone et al., 2025).

Robotic training trajectories illustrate a distinct move toward adaptive instruction. AI models individual learning curves to support personalized pathways and optimize the distribution of coaching resources (Ma et al., 2024; Moglia et al., 2022). Evidence suggests that feedback tailored to objective performance traces can enhance early skill acquisition, while predictive modeling forecasts proficiency milestones. This justifies curricula where training intensity depends on learner needs rather than fixed schedules, adhering to competency-based principles by eliminating non-informative repetition in favor of targeted practice (Ma et al., 2024; Moglia et al., 2022).

Within minimally invasive surgery, cognitive instruction focuses on perceptual guidance. Computer vision and segmentation expand training from “how to move” toward “what to recognize.” For instance, AI-assisted intraoperative navigation and 3D reconstruction in thoracic surgery enhance the identification of correct dissection planes, reducing perceptual ambiguity (Wang et al., 2025). Research into “Go/No-Go” zones and real-time anatomical labeling demonstrates that adverse events often stem from perceptual failures that trigger judgment errors (Nakao E. et al., 2025; Madani et al., 2022). By translating expert visual priorities into actionable targets—such as delineating safe regions in GI surgery—AI functions as a perceptual tutor that converts tacit expert attention into explicit criteria (Wang et al., 2025; Okumura et al., 2025; Nakao E. et al., 2025; Madani et al., 2022).

The analyzed articles also mark the emergence of language-mediated instruction as a distinct pedagogical channel. LLM-enabled support and translation systems act as synthesis interfaces that broaden global educational access and standardize content delivery (Chen et al., 2025; Becerik et al., 2025). While improving accessibility, these tools introduce unique educational risks. Concerns regarding AI hallucinations and failures in nuanced terminology may reduce learner trust and distort clinical reasoning, highlighting the need for AI literacy, verification norms, and strict curricular oversight in AI-mediated instruction (Tharakan et al., 2024; Jackson et al., 2024).

### 4.3 Comparison with other studies

Table 6 compares our systematic review with prior reviews and perspective papers that address AI in surgical training across diverse specialties and technical paradigms.

**Table 6:** Comparative summary of prior reviews and perspective studies on AI in surgical education and training, benchmarked against our systematic review.

Reference	AI application/type	Surgical area	Educational/training focus	Key findings
Nakao S. et al. (2025)	AI-assisted Robotics (PRECEYES, iArmS, OQrimo, MICRON)	Vitreoretinal Surgery (Ophthalmology)	Precision training, tremor suppression, robotic simulation, intraocular navigation	Robots improve precision in delicate surgeries. PRECEYES reached 10μm accuracy. AI supports planning and education. Tremor suppression aids training.
Sharma et al. (2025)	Augmented Reality (AR), some integration with AI	Craniofacial Surgery (Craniosynostoses)	Preoperative planning, intraoperative navigation, remote collaboration, surgical simulation	AR improves accuracy, guidance, and trainee understanding. Enhances tele-mentoring and planning. HoloLens showed accuracy improvements.
Escobar-Castillejos et al. (2025)	Machine Learning, CNN, LSTM, SVM, Clustering, etc.	Minimally Invasive Surgery, Neurosurgery, Laparoscopy, Others	Simulation platforms, Box trainers, Surgical video analysis	AI enhances personalized learning, skill assessment, and adaptive feedback. Effective in simulation setups. Challenges: heterogeneity, lack of standard metrics.
Li et al. (2024)	Network optimization, edge computing, delay-robust prediction	Telesurgery/Telementoring (General Surgery)	Remote surgical education, mentorship via telementoring	AI improves data transmission and reliability in telesurgery, enabling real-time mentoring and access in underserved regions.
Knudsen et al. (2024)	Skill assessment, intraoperative feedback, robotic autonomy	Robotic Surgery (Various Specialties)	Automated skill evaluation and real-time intraoperative training	AI analyzes surgical video and kinematics to give personalized feedback and assessments, enhancing surgical education.
Łajczak et al. (2024)	Simulation training, VR-enhanced feedback	Robotic Surgery (General/Multispecialty)	Standardized curriculum, VR simulation, dual-console training	AI-supported VR and simulators help reduce learning curves and offer personalized, immersive robotic surgery training.
Mensah et al. (2024)	Predictive analytics, machine learning models	Spine Surgery	Simulation-based training and preoperative planning	AI helps predict surgical risks and assists in planning but struggles with data quality, generalizability, and interpretability.
De Jesus Encarnacion Ramirez et al. (2024)	Augmented Reality (AR) (often paired with navigation; potential AI/ML support)	Spine surgery/Neurosurgery	Simulation, procedural rehearsal, shortening learning curves, intraoperative guidance for trainees	AR supports safer practice environments and can accelerate skill acquisition in complex spine procedures; adoption still limited by workflow, training needs, and implementation barriers.
Varghese et al. (2024)	AI/ML (deep learning, computer vision, multimodal/foundation-model direction)	Cross-specialty surgery	AI-enabled coaching, objective skill assessment, feedback loops, trainee supervision support	AI can augment apprenticeship by enabling scalable, more objective feedback and assessment from OR data (especially video); highlights risks like bias, validation gaps, and unintended training effects.
Cheikh Youssef et al. (2023)	Surgical video + AI/ML (computer vision, workflow recognition, skill scoring)	Cross-specialty (multiple procedure examples)	Video-based coaching, automated skills assessment, structured curricula using video analytics	Surgical video is positioned as core training data; AI can detect steps/actions and estimate skill to support objective assessment and targeted coaching, though performance varies and depends on data quality/labeling.
Razavi et al. (2023)	3D visualization system with AI-assisted potential for remote surgery	Ophthalmology (Vitreoretinal surgery)	Heads-up 3D displays used for telementoring and surgical education	Comparable outcomes to traditional microscopy; improved ergonomics; future potential for AI-guided teaching and global VR-based training
Georgiou et al. (2021)	5G-enabled AI for telesurgery, real-time data processing, and remote collaboration	General/Multi-specialty surgery	Telesurgical training, remote supervision, and collaborative operating environments	5G + AI reduces latency, supports real-time training, facilitates global access to surgical education and remote guidance systems
Punjani et al. (2021)	AI support in microsurgery (image processing and movement tracking)	Urology (Male infertility microsurgery)	Microsurgical training in high-precision procedures	AI may enhance surgical outcomes through improved visualization and support objective skill assessment in microsurgical education
MacLeod et al. (2025)	AI-driven imaging, robotic-assisted surgery, augmented/virtual reality tools	Spine surgery	AR/VR for surgical navigation, simulation training, robotic precision support	AI improves accuracy and training quality; AR/VR helps reduce learning curve and radiation exposure; supports real-time learning
Leivaditis et al. (2025)	AI-powered intraoperative guidance, robotics, AR systems	Thoracic surgery	Surgical planning, robotic execution, intraoperative and post-op analytics for education	Enhances accuracy, reduces complications, supports training via intraoperative decision-support; highlights ethical and implementation challenges
Constable et al. (2024)	Computer vision and markerless motion tracking using pose estimation	Cardiothoracic surgery	Objective assessment of psychomotor and cognitive surgical skills	AI enables real-time feedback on movement and coordination; supports training through data-driven performance analysis and fatigue detection
Our study	AI-enabled educational interventions, deep learning/computer vision, tutoring/predictive models, and language-based AI	Multi-domain surgical education	Characterize AI pedagogical roles, outcomes, and implementation maturity	Most studies measured performance and reported favorable effects; evidence remains early-stage with common limits from single-center designs and practical barriers.

In contrast to several studies that focus on procedure- or platform-specific innovation, our study is deliberately framed around AI as an educational intervention, emphasizing how tools are evaluated for training utility, which outcomes dominate, and why evidence maturity remains constrained.

One major distinction is specialty scope. Multiple comparative studies are anchored in narrow, high-precision domains such as vitreoretinal surgery and ophthalmic telementoring, where robotic navigation, tremor suppression, and heads-up visualization are discussed as key enablers of precision training (Nakao S. et al., 2025; Razavi et al., 2023). Similarly, craniofacial applications emphasize augmented reality for planning and intraoperative navigation, with educational value framed through improved spatial understanding and remote collaboration (Sharma et al., 2025). Our review differs in that it synthesizes a multi-domain educational landscape, where AI systems are compared across simulation, robotic and minimally invasive training, and language-mediated educational support.

A second distinction concerns the dominant pedagogical mechanism. Several comparative studies describe AI’s educational contribution primarily through technology-mediated precision and access, network optimization and edge computing to improve reliability for telementoring and underserved-region access (Li et al., 2024), or 5G-enabled infrastructures that reduce latency to support real-time telesurgical training and remote supervision (Georgiou et al., 2021). In our review, the core pedagogical lens is not infrastructure performance itself, but the training loop: objective measurement, feedback, and learner progression. This aligns more closely with cross-specialty reviews that foreground AI-enabled coaching, objective skills assessment, and feedback loops derived from OR data (Varghese et al., 2024; Cheikh Youssef et al., 2023).

Our study’s conclusions regarding evidence constraints are consistent with the broader concerns raised in the comparative literature, but we emphasize them as central limitations for educational inference. Reviews focused on simulation and video analysis repeatedly highlight heterogeneity, lack of standard metrics, and dependence on data quality and labeling factors that directly affect both model validity and the educational reliability of feedback (Escobar-Castillejos et al., 2025; Mensah et al., 2024; De Jesus Encarnacion Ramirez et al., 2024; Cheikh Youssef et al., 2023). Likewise, cross-specialty syntheses caution that scalable assessment and coaching can be undermined by bias, validation gaps, and unintended training effects, which are particularly consequential when AI outputs shape learner decision-making (Varghese et al., 2024).

The comparative table underscores an expanding frontier in which AI supports cognitive and ergonomic dimensions of training. Motion tracking and pose estimation are presented as routes to real-time feedback, fatigue detection, and objective assessment in cardiothoracic settings (Knudsen et al., 2024; Constable et al., 2024), while thoracic surgery-focused work highlights intraoperative decision support and ethical/implementation challenges that accompany training benefits (Leivaditis et al., 2025). These perspectives complement our synthesis by reinforcing that future effectiveness should be defined beyond accuracy metrics—incorporating workflow integration, safety, and governance while maintaining rigorous educational endpoints.

In summary, compared with specialty- and platform-focused reviews (Nakao S. et al., 2025; Sharma et al., 2025; Łajczak et al., 2024), our study provides a cross-domain educational synthesis, aligning with evidence that positions AI as a scalable coaching and assessment layer (Varghese et al., 2024; Cheikh Youssef et al., 2023), while highlighting persistent standardization and implementation barriers emphasized across the broader literature (Escobar-Castillejos et al., 2025; Georgiou et al., 2021).

### 4.4 Limitations

The analyzed articles should be interpreted in light of several constraints inherent to the rapidly evolving field of AI-enabled surgical education. A primary limitation is the marked heterogeneity in how educational impact was defined and measured. The reviewed studies used endpoints ranging from objective technical metrics and algorithmic accuracy to knowledge tests and perception surveys, which limited cross-study comparability and precluded estimation of a consistent magnitude of educational benefit across surgical modalities and settings. Reporting also emphasized short-term improvements in controlled environments, which may not reflect sustained learning, skill retention, or transfer to clinical practice. The current evidence base may also be weighted toward favorable findings. Relatively few studies reported neutral or negative effects, raising concerns about publication and selective reporting bias, particularly in development-oriented research in which early feasibility is often prioritized. Accordingly, the overall direction of effect should be interpreted as encouraging rather than as definitive evidence of broad educational effectiveness. In addition, no formal risk-of-bias or study quality assessment tool was applied, which further limits confidence in cross-study comparisons and in the overall strength of the conclusions.

Evaluation strategies also remained predominantly performance-centered. Most studies prioritized measurable competence proxies such as speed, accuracy, error rates, and proficiency classification, especially in simulation, XR/VR, and robotic platforms. By contrast, outcomes related to engagement, adoption, and implementation were evaluated less frequently and often relied on subjective self-report measures. This imbalance limits insight into whether AI tools are appropriately trusted, sustained within existing curricula, or equitably applicable across learner populations and resource-constrained settings. Interpretation is further complicated by the coexistence of heterogeneous study types, including technical development and validation studies, active training interventions, and readiness- or acceptability-focused studies. Because these categories address different educational questions, they are not directly comparable and should not be interpreted as equivalent forms of evidence regarding educational effectiveness.

Evidence maturity is further constrained by the early-stage nature of many contributions. A substantial portion of the literature relies on single-center designs, small samples, and brief exposure periods in nonclinical environments, reducing external validity and limiting inference regarding real-world instructional value in the operating room. Sparse longitudinal follow-up further weakens conclusions about the durability of acquired skills. Practical and ethical barriers also affect interpretability and scalability. Variability in video capture, anatomical complexity, and visual occlusion can reduce model robustness, while hardware cost and the absence of haptic feedback may hinder broader deployment. Language-mediated tools introduce additional concerns, including semantic errors and unreliable generation, which may affect learner reasoning if outputs are not rigorously verified. Privacy and governance also remain critical considerations, particularly when AI is integrated into high-stakes educational decision-making or applied to sensitive clinical data.

## 5 Conclusion

This systematic review indicates that artificial intelligence is being used across a growing range of applications in surgical education and training. Across the included studies, AI-based tools were most often applied to objective feedback, performance assessment, task recognition, simulation-based learning, and selected components of clinical reasoning. These findings suggest that AI may support specific aspects of surgical education, particularly when linked to measurable outcomes such as technical proficiency and structured formative assessment. The evidence, however, remains heterogeneous and is still dominated by early-stage development, restricted educational deployment, and short-term evaluation rather than routine curricular integration. In addition, the absence of a formal risk-of-bias or study quality assessment limits confidence in the comparative interpretation of the included studies.

Although several studies reported favorable results in controlled or simulation-based settings, the current literature supports feasibility and early educational value more strongly than sustained educational adoption. These findings should not be interpreted as evidence of direct clinical benefit, since most included studies evaluated technical, educational, or simulation-based outcomes rather than patient-level or real-world operative endpoints. Future progress will depend on prospective multicenter studies, clearer outcome standardization, formal quality appraisal, and stronger evaluation in real educational settings to define the role of AI within competency-based surgical education.

## Data Availability

The original contributions presented in the study are included in the article/Supplementary material, further inquiries can be directed to the corresponding author.
