# Supplementary material for: Artificial intelligence applications in surgical education and training: a systematic review
Source: Front Artif Intell. 2026 Jun 24;9:1815315. doi: 10.3389/frai.2026.1815315 (PMC13343274; doi:10.3389/frai.2026.1815315)
Supplement: Supplementary file 1 [file Supplementary_file_1.docx]

Scheme Prompts

SCHEME 1 PROMPT:
[Title]
At the top-center of a clean white background, place the main title in bold, dark blue text: "DATABASES SELECTED FOR THE REVIEW". Below this, in a slightly smaller, dark grey font, add the subtitle text: "Healthcare | Education | Technology".
[Structure]
Create a three-column infographic layout. Each column is a distinct vertical module with a unique color scheme for its top section and bullet point icons. All columns share a unified layout: a top text header, a central visual illustration, a descriptive list, and a small bottom text tagline. The overall style is clean, academic, and uses modern, stylized graphics.
[Left Column - Scopus]
Header: The background is a soft beige color. The main text header, in large bold dark blue font, is "Scopus". Below it, the secondary text is "Scopus®".
Central Visual: A desk scene with a stack of old leather-bound books and a black optical microscope. The background shows a light-filled workspace with more books and paper.
Descriptive List: Two text points with orange gear icons.
Point 1: "Broad Indexing of Peer-Reviewed Journals"
Point 2: "Health Sciences, Education & Engineering"
Tagline: (Not visible in image).
Column Background: White below the beige header.
[Center Column - PubMed]
Header: The background is a soft blue color. The main text header, in large bold dark blue font, is "PubMed".
Central Visual: A stylized, circular composition with a DNA helix, an open medical book (with images of the human heart and brain), a smiling male doctor in a white coat holding a tablet, and digital medical icons (like a heart monitor) floating around him. The background is a clean, circular light blue.
Descriptive List: Two text points with green caduceus icons.
Point 1: "Biomedical Literature"
Point 2: "Clinical & Healthcare Education Research"
Tagline: (Not visible in image).
Column Background: White below the blue header.
[Right Column - IEEE Xplore]
Header: The background is a soft green color. The main text header, in large bold dark blue font, is "IEEE Xplore".
Central Visual: A robotic arm lifting an "AI" processor chip from a complex motherboard with circuits. In the background are more schematic circuit diagrams on a wall-mounted panel. The style is detailed and tech-focused.
Descriptive List: Two text points with green "IC" (Integrated Circuit) chip icons.
Point 1: "AI & Engineering Research"
Point 2: "Tech & Simulation Hardware"
Tagline: (Not visible in image).
Column Background: White below the green header.
[Footer]
At the bottom of the entire image, centered, add the final tagline in two lines of dark grey text:
"Comprehensive & Multidisciplinary Coverage" (in a bold font)
"Healthcare, Pedagogy & Computational Technologies" (in a slightly smaller, normal font)
[Final Styling Notes]
Lighting: Bright, clean, studio-lit look.
Typography: Clear, professional, sans-serif fonts throughout. Use bolding as specified for hierarchy.
Icons: Use the specific icons (gears, caduceus, IC chips) with their correct colors.
Overall Feel: Crisp, organized, and professional, like a high-quality academic presentation slide. The colors should be soft yet distinct.

SCHEME 2 PROMPT:
A medical schematic diagram on a clean white background, detailing the evidence maturity pathway for AI in surgical education and training. The diagram features a single, long horizontal bar that progresses from a light gray-blue color on the left to a deep reddish-orange color on the right. Small black hash marks act as separators on the bar. Above the bar are three distinct stations, each with its own icon and color-coded arrow pointing down. The first station on the left has icons of a beaker of green liquid and a set of surgical gloves and mask, labeled with the text "Development & Simulation", and a green arrow. The central station has an icon of a clipboard with a question mark and a small red question mark badge, labeled "Curriculum Integration?", and an orange arrow. The final station on the right has an icon of a multi-story hospital building with a question mark above it, labeled "Real-World Outcomes?", and a red arrow. Below the bar, small-text labels "SCHEME 2" and "Evidence maturity pathway for AI in surgical education and training." are present. The overall style is clean and informative.

SCHEME 3 PROMPT:
"A complex, professional, diagram of a "Pedagogical trajectory of AI-enabled surgical education," The diagram is divided into three distinct vertical zones (triptych-like, with subtle background room shifts, but clean overall). A prominent, wide, arcing orange-to-green flow arrow frames the top with the text "Retrospective Feedback".
Zone 1 (Left, orange-tinted background, like a data lab): Large brown text "Measurement". Below it is a clean computer monitor displaying intricate, colorful bar charts and line graphs showing performance metrics. This zone has a warm, data-driven light.
Zone 2 (Center, cool blue-tinted background, like a simulation room): Large blue text "Guided Practice". An adjacent monitor (angled similarly to Zone 1's) shows a highly-detailed, photorealistic simulation: a surgical robotic hand (with delicate steel linkages) is performing a precise procedure on a complex, synthetic organ (e.g., a vascular structure) inside a simulator cavity. This area is lit with precise, surgical blue light.
Zone 3 (Right, white/light green background, like an AI learning hub): Large green text "Adaptive Instruction". This area has an advanced, high-tech cerebral-profile-like structure with an artificial brain cortex (showing active neural pathways) and a stack of digitized-looking books with small, intricate floating 'A' and translated characters, indicating language model adaptation. This area is brightly lit with clean, intellectual light.
The text below the image is perfectly legible, in two lines:
"SCHEME 3
Pedagogical trajectory of AI-enabled surgical education."
The overall style is clean, modern, and highly polished, using a cohesive color palette that transitions from orange to cool blue to green, signifying the learning stages. The background environments (data lab, simulator room, AI hub) are implied through lighting and subtle depth. The transition between the monitors and the AI head is seamless and organized."
